# Automatic monitoring of plateau and driving pressure during pressure and volume controlled ventilation

**DOI:** 10.1186/2197-425X-3-S1-A998

**Published:** 2015-10-01

**Authors:** F Mojoli, M Pozzi, S Bianzina, G Tavazzi, A Orlando, S Mongodi, F Torriglia, A Braschi

**Affiliations:** Anesthesia and Intensive Care, Fondazione IRCCS Policlinico S. Matteo, University of Pavia, Pavia, Italy

## Introduction

Plateau pressure (Pplat) limitation is routinely used to avoid ventilator-induced lung injury. Recently, driving pressure (ΔP) was strongly associated with survival in ARDS patients [1].

## Objectives

To evaluate the feasibility of ΔP and Pplat continuous monitoring during volume (VCV) and pressure (PCV) controlled ventilation, we compared with gold standard (occlusion maneuvers at end-inspiration and end-expiration) two different methods: 1- Least Square Fitting (LSF) method that provide maneuver-free Pplat and ΔP values; 2- Mini Occlusion (MO) method by performing brief occlusion maneuvers.

## Methods

We enrolled 22 patients admitted to our ICU after scheduled major abdominal surgery, with normal or restrictive respiratory system mechanics under pressure (12 pts) and volume (10 pts) controlled mechanical ventilation (G5, Hamilton Medical). We studied 12 different conditions in each patient by changing respiratory rate (10-15-20-25 bpm) and I:E ratio (1:2, 1:1, 2:1). Inspiratory pressure in PCV and tidal volume (TV) in VCV were adjusted to keep end-tidal CO_2_ between 32 and 36 mmHg. PEEP and FiO_2_ were set to maintain SpO_2_ ≥ 95%. ΔP, Pplat and PEEPtot reference values (ΔP_REF_, Pplat_REF_ and PEEPtot_REF_) for each ventilatory setting were obtained by 5s-length occlusion maneuvers at end-inspiration and end-expiration (ΔP_REF_ = Pplat_REF_ - PEEPtot_REF_). ΔP_MO_ values were calculated as: Pplat_MO_ - PEEPtot_MO_, Pplat_MO_ and PEEPtot_MO_ being average airway pressure in the last 100 ms of mini-occlusion maneuvers lasting 400 ms. LSF applied over the whole respiratory cycle provided Pplat_LSF_ and Crs_LSF_ values, being Crs the compliance of the respiratory system. ΔP_LSF_ was calculated as TV/Crs_LSF_.

## Results

Difference with reference values was greater for MO vs. LSF, both for ΔP (0.82 ± 0.41 vs. 0.46 ± 0.82 cmH_2_O, respectively; p < 0.001) and Pplat (0.52 ± 0.33 vs. 0.02 ± 0.49 cmH_2_O; p < 0.001).

## Conclusions

Both methods provided a good estimation of ΔP and Pplat; MO showed a slightly better precision than LSF, but a greater bias, being these differences not clinically relevant. Anyway, LSF is a totally continuous and non invasive method, whereas MO method minimally interferes with mechanical ventilation and its implementation on ICU ventilators could be more troublesome.
